# Pre‐Hospital Delay in STEMI: A Narrative Review of Symptom Onset–to–First Medical Contact Across High‐Income and Low‐ and Middle‐Income Countries

**DOI:** 10.1002/clc.70352

**Published:** 2026-05-22

**Authors:** Javeria Akhter, Zain Ul Abedin, Huzafa Ali, Ahmed Khan, Maryam Nasir, Eeman Afroz, Muhammad Noshab

**Affiliations:** ^1^ Indus Hospital and Health Network Karachi Pakistan; ^2^ King Edward Medical University Lahore Pakistan; ^3^ CMH Multan Institute of medical Sciences Multan Pakistan; ^4^ Peshawar Medical College Peshawar Pakistan; ^5^ Ivane Javakhishvili Tbilisi State University Tbilisi Georgia; ^6^ Allama Iqbal Medical College Lahore Pakistan; ^7^ International University of Kyrgyzstan Bishkek Kyrgyzstan

**Keywords:** emergency medical services, first medical contact, health systems, pre‐hospital delay, STEMI

## Abstract

**Aim:**

ST‐segment elevation myocardial infarction (STEMI) remains a major contributor to global cardiovascular morbidity and mortality, with outcomes varying considerably between high‐income and low‐ and middle‐income countries (LMICs). Although advances in reperfusion therapy and improvements in hospital‐based performance have decreased in‐hospital delays, pre‐hospital delay remains one of the largest components of total ischemic time, thereby restricting the survival benefit of timely reperfusion.

**Methods:**

This narrative review synthesizes evidence on pre‐hospital delay in STEMI and interprets delay through a health‐system performance and equity lens rather than viewing it only as an individual behavioral issue. Relevant studies were identified through a search of PubMed, Embase, and Scopus (2000−2025). Evidence was interpreted using a conceptual framework comprising four pathways through which pre‐hospital delay is produced: recognition, decision‐making, access, and system readiness.

**Results:**

Across multiple observational studies and registry reports, median symptom onset–to–first medical contact intervals in high‐income countries are typically 60–120 min, supported by organized STEMI networks and higher EMS utilization, although inequities persist among vulnerable population. In several LMIC settings, symptom onset–to–FMC delays frequently exceed 300 min and may extend beyond 480 min, driven by limited EMS coverage and fragmented referral pathways.

**Conclusion:**

Overall, pre‐hospital delay in STEMI is a multidimensional and inequitable phenomenon suggesting the need for integrated approaches beyond awareness campaigns, including EMS strengthening, coordinated referral networks, early diagnostic capacity, financing protections, and governance and accountability mechanisms. Positioning emergency cardiac care within universal health coverage and national non‐communicable disease strategies may reduce avoidable STEMI mortality and narrow global inequities.

## Graphical abstract

Pre‐hospital delay in STEMI is driven by interacting patient, social, and health‐system factors rather than individual behavior alone. Addressing delays requires system‐level interventions, including strengthening emergency medical services, improving early diagnosis, and integrating coordinated STEMI care within universal health coverage frameworks.

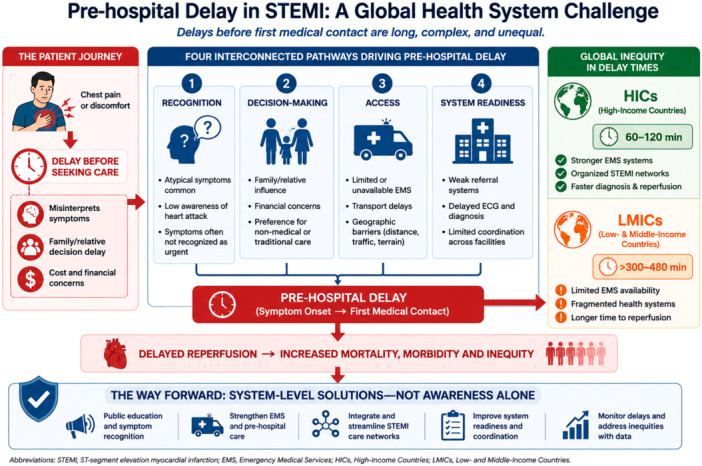

## 1 Introduction

### 1.1 The Time‐Critical Nature of STEMI

ST‐segment elevation myocardial infarction (STEMI) remains a major contributor to cardiovascular mortality and morbidity, accounting for more than 3 million cases annually worldwide [1]. While advances in reperfusion therapy and emergency care have noticeably improved outcomes, these improvements have been disproportionately distributed across the globe. In high‐income countries (HICs), in‐hospital deaths have reduced to 5%–6% due to the establishment of organized STEMI networks and standardized reperfusion protocols [2], but in many low‐ and middle‐income countries (LMICs), the mortality rate remains approximately 9%−10% or higher because of fragmented health systems, delayed presentation, and limited access to timely reperfusion [3].

These outcome differences are closely linked to the time‐sensitive nature of myocardial recovery. The benefit of reperfusion therapy decreases with an increase in treatment delay. Observational studies have reported that each 30‐min delay in reperfusion is associated with an increase in relative mortality risk, underscoring the time‐sensitive nature of STEMI care [4]. Despite improvements in hospital‐based system performance metrics such as ECG acquisition within 10 min of FMC and FMC‐to‐device time, pre‐hospital delay remains one of the largest components of total ischemic time [5].

In HICs, EMS is used by approximately 60%–75% of STEMI patients as the primary transport mode in structured registry settings, whereas in many LMIC contexts, formal EMS is used by fewer than 25%–30% of patients, with the majority relying on private vehicles or informal transport [6, 7]. These infrastructure differences translate directly into reperfusion inequity: reperfusion rates consistently exceed 85%–90% in well‐resourced HIC settings with organized STEMI networks, compared with rates below 50%–65% in numerous LMIC registries [6, 8].

### 1.2 Defining Pre‐Hospital Delay

Pre‐hospital delay refers to the time interval from the onset of symptoms to the first medical contact (FMC), defined here as the first assessment by trained healthcare personnel capable of obtaining and interpreting an electrocardiogram (ECG) and initiating appropriate management. To avoid definitional overlap, this review distinguishes (i) symptom onset–to–FMC (pre‐hospital delay) from (ii) FMC–to–diagnosis (e.g., ECG acquisition/interpretation) and (iii) FMC–to–device (wire crossing) or arrival at a PCI‐capable center, which capture post‐contact system delays. Delayed ECG acquisition, mis‐triage, and referral inefficiencies are therefore considered as post‐contact system delays to reperfusion rather than components of pre‐hospital delay [9].

Nevertheless, the magnitude and determinants of pre‐hospital delay vary greatly across health systems and socioeconomic contexts, highlighting differences in emergency access and system organization. Across many HICs registry and observational reports, such as in the US and Europe, median symptom onset–to–FMC intervals are often reported in the range of approximately 60–120 min [10], whereas studies from several LMIC settings, including India, China and South Africa, report delays often exceeding 300–480 min [11]. These differences can translate into substantially longer total ischemic time and are likely to contribute to outcome disparities, although the magnitude varies by setting, pathway, and study design.

Referral system architecture further distinguishes HIC and LMIC contexts: most HICs operate designated spoke‐and‐hub STEMI networks with formal inter‐facility transfer protocols and pre‐hospital catheterization laboratory activation, whereas in many LMICs, hospitals are not formally classified as PCI‐capable or non‐PCI‐capable, and referral pathways remain informal and inconsistent, substantially prolonging the interval from initial presentation to definitive reperfusion [8, 12].

### 1.3 Limitations of Existing Literature

Although the literature on pre‐hospital delay in STEMI is extensive, existing work is uneven in scope. The majority of the studies focus primarily on individual behaviors such as attribution of symptoms and lack of awareness, while fewer examine health system determinants like EMS access, referral processes, financing barriers, or delay after FMC. Additionally, equity dimensions, such as gender, geography, and socioeconomic status, are inconsistently reported across registries and observational studies [10]. This evidence gap restricts the design and implementation of the interventions that address the structural and policy‐level causes of pre‐hospital delay.

### 1.4 Emergence of a Public Health Perspective

Over the past decade, there has been growing recognition that emergency cardiac care is not only a clinical response, but a fundamental public health responsibility. Effective management of STEMI in the pre‐hospital phase depends not only on individual behavior but also requires access to emergency medical services (EMS), well‐organized local coordination, and supportive policy frameworks that enable rapid diagnosis and treatment [13].

Yet, profound inequities continue worldwide. More than 80% of global cardiovascular deaths occur in LMICs, where fragmented health systems, financial barriers, and geographic disparities often restrict access to life‐saving interventions [14]. In many of these settings, less than 65% of STEMI patients receive any form of reperfusion therapy in comparison to more than 90% in most of the HICs [6].

These differences highlight that pre‐hospital delay is not merely a matter of patient awareness, but a reflection of health system design and policy priorities. Reframing emergency cardiac care as a critical component of universal health coverage (UHC) and the non‐communicable disease (NCD) agenda underscores the need for integrated, equity‐oriented system strengthening. Viewed through a public health lens, delays in STEMI care can be understood as indicators of health‐system performance and equity, highlighting how policy choices, financing, and emergency‐care capacity shape time‐to‐treatment.

Over the last two decades, some LMICs have made measurable progress: regional STEMI pilots in Tamil Nadu (India), Salvador (Brazil), and Jakarta (Indonesia) have demonstrated meaningful improvements in reperfusion rates through hub‐and‐spoke models combined with telemedicine and pharmaco‐invasive strategies [3, 7, 15]. Nevertheless, these gains remain geographically and institutionally concentrated, and have not yet translated into sustained national‐scale improvements in most settings, underscoring the need to move from pilot demonstration to systemic health system reform.

### 1.5 Rationale

The previous discussion highlights that pre‐hospital delay in STEMI is shaped by the organization, financing, and governance of health systems. Yet, the existing evidence remains inadequate to guide system‐level action, especially in LMICs. Although global research has extensively measured pre‐hospital delays, less progress has been made in understanding their structural determinants across different health system contexts. Therefore, policy responses often emphasize public awareness or symptom education, while neglecting system‐level barriers that shape patient pathways to FMC.

Simultaneously, interventions such as pre‐hospital ECG activation, regional STEMI networks, and 24/7 PCI centers have proven highly effective in HICs, but their implementation has yielded mixed or unsustained results in LMICs due to contextual constraints related to financing, workforce capacity, and infrastructure.

Therefore, there is a need for a systems‐oriented synthesis that moves beyond individual explanations of health‐seeking behavior. By considering insights from clinical medicine, health policy, and social science, this review aims to:

- Illustrate the structural and contextual factors driving delay across settings;
- Identify strategies suitable for implementation in a resource‐constrained setting; and
- Provide a conceptual framework for positioning STEMI care with wider goals of UHC and NCD prevention.

In doing so, this review moves beyond measuring the duration of delay, toward understanding the system‐level drivers of delay, the role of health‐system readiness, and potential strategies to reduce these delays equitably.

## 2 Methods

This study was conducted as a narrative review synthesizing evidence on pre‐hospital delay in STEMI across HICs and LMICs. The search strategy, study selection criteria, country classification, and synthesis approach are described in detail in Section 2.1.

### 2.1 Literature Search and Selection

Literature was identified through structured searches of PubMed, Embase, and Scopus covering publications from 2000 to 2025. Search terms included combinations of: (“STEMI” OR “ST‐elevation myocardial infarction”) AND (“pre‐hospital delay” OR “symptom onset to first medical contact” OR “patient delay”) AND (“emergency medical services” OR “EMS”); further combined with terms for “referral networks,” “reperfusion therapy,” “low‐ and middle‐income countries,” and “health system.” A representative PubMed search string was: (“STEMI” OR “ST‐elevation myocardial infarction”) AND (“pre‐hospital delay” OR “symptom onset to first medical contact” OR “patient delay”) AND (“emergency medical services” OR “EMS”) AND (“referral networks” OR “reperfusion therapy” OR “health system” OR “low‐ and middle‐income countries”). Similar adaptations of this strategy were applied across Embase and Scopus. This narrative review aimed to capture representative and influential studies across diverse geographic and health‐system contexts rather than provide exhaustive retrieval. Additional articles were identified through citation tracking of key reviews, clinical guidelines, and major registry reports.

The review focused exclusively on peer‐reviewed publications; conference abstracts and preprints were excluded to preserve evidential quality. Countries were classified as high‐income or low‐ and middle‐income according to the World Bank income classification current at the time of study publication. Because of substantial heterogeneity in study designs, delay definitions, and outcome measures, evidence was synthesized narratively using a four‐pathway conceptual framework (recognition, decision‐making, access, and system readiness). Deliberate efforts were made to include studies from diverse geographic regions—including sub‐Saharan Africa, South and East Asia, Latin America, and the Middle East—to reduce geographic selection bias. Nevertheless, the inherent subjectivity of narrative review means that study selection and synthesis reflect author judgment, and this should be considered when interpreting conclusions.

## 3 Evidence Synthesis: Pathways Contributing to Pre‐Hospital Delay in STEMI

The findings from the reviewed literature were synthesized narratively using a conceptual framework describing four pathways contributing to pre‐hospital delay in STEMI. Evidence from different studies were organized within these domains to illustrate how patient‐level, social and health‐system factors interact to influence symptom onset–to–FMC timelines across settings.

### 3.1 Recognition Pathway

Recognition of symptoms is a key determinant of pre‐hospital delay in STEMI. Patients often experience a mismatch between expected and actual symptoms, with many anticipating classic chest pain or radiating arm and shoulder pain but instead experiencing nausea, dizziness, shortness of breath, or feverish sweats [16]. Such atypical symptoms are frequently misattributed to gastrointestinal causes, which contributes to prolonged delay before seeking care [17, 18]. Atypical presentations are particularly common among older adults, women, and patients with comorbidities such as diabetes or chronic kidney disease, and these presentations are associated with longer pre‐hospital delay and higher mortality [19, 20]. Age appears to be a stronger predictor than sex for atypical presentation, with the proportion of atypical symptoms rising from 11% in patients under 55% to 51% in patients over 85 [21, 22].

Prior experience with cardiac disease also shapes the urgency of care‐seeking behavior. Patients with a family history of MI or coronary artery disease demonstrate shorter pre‐hospital delays and seek care more promptly [23, 24, 25]. Patients with cardiogenic shock, previous MI, or familial history of ischemic heart disease are more likely to recognize symptoms quickly and activate emergency services [25, 26]. Conversely, patients without prior cardiac experience are more likely to misattribute symptoms to non‐cardiac causes, including age‐related misinterpretation [27].

The combination of symptom misinterpretation, atypical presentations, and prior cardiac experience defines the recognition pathway and constitutes a major element of pre‐hospital delay in STEMI.

### 3.2 Decision‐Making Pathway

Pre‐hospital delay in STEMI is strongly influenced by family‐mediated decision‐making. Patients who are away from home or have a bystander present tend to seek care more quickly than those who are alone, highlighting the protective effect of immediate social support [10, 28, 29]. The presence of a spouse or family member also contributes to shorter decision times, with married patients generally experiencing reduced delays compared to unmarried individuals [10, 30].

Gender and power dynamics within households further shape the decision‐making process. Men's delays are often influenced by concerns about financial responsibility and family maintenance, whereas women may delay seeking care due to worries about the well‐being of their children and adherence to social roles and expectations [31, 32]. Women also face delays related to social factors, such as reliance on attendants or family members, while men's delays are more often linked to individual interpretations of symptoms [31]. Behavioral factors, including hesitation in responding to symptoms and the presence of atypical presentations, also contribute to delay, though the presence of bystanders consistently reduces delay for both men and women [33]. Stereotypical beliefs about gender roles do not appear to directly determine help‐seeking behavior, indicating that socialized norms interact with individual experiences and situational contexts in complex ways [34].

Anticipated costs and affordability concerns are additional determinants of pre‐hospital delay. Patients who are uninsured or concerned about potential treatment costs often delay seeking care [35], whereas financial barriers appear less influential in some populations, suggesting variability based on context [36].

### 3.3 Access Pathway

Access‐related factors play a critical role in shaping pre‐hospital delay in STEMI, particularly through the availability and reliability of EMS. Temporal factors influence access to care, with symptom onset during nighttime hours associated with longer delays in calling EMS compared to daytime onset [37]. Mode of transport further affects timeliness, as patients transported by EMS generally reach FMC more rapidly and experience shorter overall treatment timelines. Shorter EMS‐related delays are also associated with improved survival outcomes, underscoring the importance of efficient pre‐hospital systems [38].

Patterns of EMS utilization remain suboptimal in many settings. A study conducted by Yang et al. in China reported that only a minority of STEMI patients used EMS, with most patients opting for private or self‐transport, despite EMS use being associated with shorter hospital delays [39]. Similar trends have been observed elsewhere, with less than half of STEMI patients arriving by ambulance and the remainder relying on private vehicles [40]. Use of EMS is more likely among women, elderly patients, individuals of higher socioeconomic status, and those with a prior history of ischemic heart disease [41]. Symptom characteristics also affect access behavior, with classical chest pain accompanied by stress‐related symptoms increasing the likelihood of EMS activation compared to isolated or less alarming presentations [42].

Geographic constraints further modulate access to timely care. Rural residence is associated with longer EMS response, on‐scene, and transport intervals compared to urban settings, resulting in a lower likelihood of early FMC [43]. Evidence from systematic reviews indicates that EMS performance is generally superior in urban areas, where response times and transport efficiency are more favorable, highlighting persistent urban–rural disparities in access to emergency cardiac care [44].

### 3.4 System Readiness Pathway

Although these processes primarily occur after FMC, they are included because system readiness influences how effectively early presentation translates into definitive treatment. Organized, out‐of‐hospital triage–based STEMI networks have been shown to reduce short‐term fatality as well as long‐term mortality, highlighting the value of coordinated referral pathways that activate definitive care early [45]. Timely triage remains critical because primary percutaneous coronary intervention must be delivered within a narrow therapeutic window to reduce mortality, yet a substantial proportion of patients fail to meet this benchmark, reflecting limitations in system capacity rather than patient behavior alone [46].

The diagnostic capability at FMC is another key component of system readiness because it determines the speed of FMC‐to‐diagnosis and FMC‐to‐reperfusion intervals. Introduction of a medical priority dispatch system for acute coronary syndromes (ACS) in China was associated with improved pre‐hospital care, higher diagnostic accuracy at FMC, and shorter intervals from emergency call to EMS arrival [47]. Pre‐hospital ECG acquisition and interpretation demonstrate good diagnostic accuracy overall, supporting their role in early STEMI identification and shortening post‐contact system delays [48].

**Figure 1:**
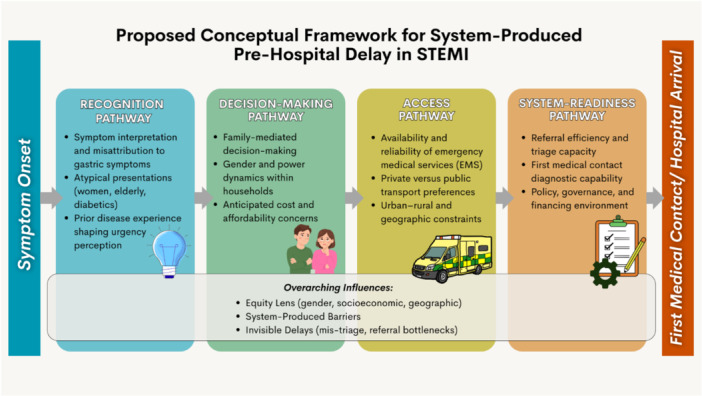
Proposed conceptual framework for system‐produced pre‐hospital delay in STEMI. This framework reframes pre‐hospital delay as a multi‐pathway, system‐generated process shaped by equity and structural determinants, rather than solely individual patient behavior.

Additionally, broader policy, governance, and financing environments shape the effectiveness of these system‐level processes. Governance has been identified as a frequently overlooked yet critical determinant of pre‐hospital emergency care performance, influencing coordination, equity, and the timely delivery of services. Weak legislative and governance frameworks may contribute to fragmented pre‐hospital systems, particularly in LMIC settings, thereby constraining rapid access to definitive care [49]. System‐level reviews consistently emphasize that high‐quality pre‐hospital STEMI care depends on integrated emergency care systems, including early ECG acquisition, telemedicine support, clinical decision tools, and sustained EMS training, rather than isolated time‐based metrics alone [50] (Figure 1).

## 4 Evidence From HICs

### 4.1 Magnitude and Trends in Delay

Evidence from high‐income settings suggests that delays across the STEMI care pathway have declined over time, reflecting improvements in EMS and reperfusion pathways. Longitudinal data from an EMS system in Florida demonstrate improvements in system performance after FMC, including increasing proportions of patients achieving recommended EMS‐to‐balloon benchmarks [51]. Similarly, a prospective cohort study from Sweden reported a reduction in overall treatment delays across the STEMI care pathway, underscoring the impact of structured STEMI networks and standardized care pathways.

Despite these overall improvements, residual delays persist even within advanced healthcare systems. In the Swedish cohort, women, older adults and patients with diabetes continued to experience longer delays compared to other patient groups, highlighting enduring disparities that have not been fully addressed by system‐level advances alone [29]. Comparative evidence further illustrates these patterns across income settings. In a study contrasting centers in a HIC and an LMIC, most patients in the high‐income setting used EMS and reached the FMC more rapidly, whereas patients in the lower‐resource setting predominantly relied on private transport, such as taxis, and arrived substantially later after symptom onset [52].

### 4.2 Role of EMS and Early Diagnostic Capacity

Early diagnostic interventions after FMC, including rapid ECG acquisition and pre‐activation of the catheterization laboratory, play a critical role in reducing system delay and improving outcomes in STEMI. In Australia, pre‐hospital activation of the Cath lab was associated with shorter FMC‐to‐reperfusion intervals and improved mortality, demonstrating the impact of early system‐based interventions on patient outcomes [53]. Similarly, pre‐hospital ECG interpretation, whether performed in the ambulance or transmitted to expert centers, has been shown to provide accurate diagnostic information and facilitate early treatment decisions [48]. In the United Kingdom, use of pre‐hospital ECG acquisition for suspected ACS increased the odds of timely reperfusion and was linked to improved survival, further highlighting the value of early diagnostic capability [54].

Despite these advances, delays persist among patients who do not utilize EMS and instead self‐present or are transferred from other facilities, even in HICs, indicating that pre‐hospital diagnostic strategies after FMC primarily improve outcomes for patients who engage with emergency services [55].

### 4.3 Persistent Inequities

Even in HICs, disparities in pre‐hospital delay remain evident across patient subgroups. Although historical data indicate that women previously experienced substantially longer delays, more recent evidence from Switzerland suggests that this gap has narrowed, highlighting the potential impact of targeted improvements over time [56]. Socioeconomic factors also contribute to differences in pre‐hospital care, with individuals of lower socioeconomic status at risk of experiencing disparities in access to or utilization of emergency services [57].

### 4.4 Lessons From HIC Settings

Experience from HICs suggests that reducing STEMI delay requires coordinated systems of care rather than isolated technological improvements. The main lesson from HIC settings is that patient access, EMS activation, and post‐contact diagnostic efficiency perform best when embedded within coordinated regional systems of care. However, persistent delays among women, older adults, and socioeconomically disadvantaged groups indicate that system optimization alone does not eliminate inequity in early presentation [52]. These findings suggest that improvements in system performance must be complemented by strategies addressing patient‐level and social determinants of delay.

## 5 Evidence From LMICs

Timely reperfusion in STEMI, particularly within the first hours after symptom onset, is associated with improved myocardial salvage, preservation of left ventricular function, and lower mortality [52]. Thrombolytic therapy is most effective when administered within the first hour after symptom onset (“the golden hour”), although clinical benefit may extend for several hours after symptom onset, depending on context and treatment strategy [25]. However, in the majority of LMICs, the interval between symptom onset and treatment initiation is often much longer than necessary for optimal or efficient ACS care, which remains a challenging problem. Early presentation delays remain substantial in Bangladesh and similar LMIC settings; less than 40% of patients appear within the crucial 6‐h window, and median early presentation intervals (often measured as symptom onset to hospital arrival) exceed approximately 9 h, suggesting prolonged delays before FMC [25]. Nearly two‐thirds of STEMI patients in a prospective cohort study from Karachi, Pakistan, presented to a healthcare facility more than 6 h after symptom onset, indicating prolonged delays before FMC [58]. Furthermore, a meta‐analysis of LMICs showed marked pre‐hospital delays in STEMI, with mean early presentation intervals (often measured as symptom onset to FMC) of approximately 4.7 h, increasing to nearly 8 h, far exceeding recommended reperfusion windows [59]. Numerous factors contribute to these delays, with the absence of organized regional STEMI care systems remains a major structural barrier to consistent management, reflecting limited financial, infrastructural, and human resources. This is compounded by underdeveloped EMS and poorly equipped emergency departments for ACS care [60]. Due to inadequate emergency services and poor transportation, rural populations in LMICs often have limited access to PCI‐capable centers, which are typically concentrated in urban areas. Timely care is further hampered by a limited ambulance availability, shortages of trained paramedics, and inconsistent policy support. Help‐seeking is delayed by low health literacy and a lack of knowledge about STEMI symptoms, which frequently results in patients seeing unqualified clinicians. Additionally, most patients’ access to modern cardiac care is limited by insecure employment, high treatment expenses, and lack of insurance coverage [8]. Patients must deal with the challenges of costs and payments at a particularly sensitive moment, while also navigating barriers at different levels of the health system. In LMICs, third‐party insurance and government‐sponsored plans are usually nonexistent or very basic, and a large share of medical expenses is paid at the point of care in cash or cash equivalents. Such out‐of‐pocket payments can create barriers even for relatively affluent patients presenting with STEMI [61]. Gender disparities also remain evident in STEMI care, as women in LMICs experience poorer outcomes compared to men due to delayed hospital presentations, underutilization of evidence‐based therapies, and high burden of comorbidities. For example, according to a study done in Egypt with 400 ACS patients, women experienced much worse in‐hospital outcomes than men, with a greater rate of MACE and cardiac death [61]. Furthermore, age, race/ethnicity, and geography significantly influence treatment and outcomes. Older patients (≥ 85 years) have substantially higher mortality than younger groups and are considerably less likely to receive evidence‐based reperfusion or PCI, in part because of atypical presentations and undertreatment. Similar disparities related to age, ethnicity, and geography have been reported across multiple health systems [62].

## 6 Comparative Analysis: Why Delays Persist

The majority of LMICs have experienced an epidemiological shift over the last few decades from endemic infectious and nutritional disorders to NCDs in general and cardiovascular disease in particular. As a result of this transition, many LMIC health systems face significant implementation and capacity gaps in the management [63]. In high‐income nations, the use of guideline‐directed therapies has significantly improved STEMI outcomes. To optimize management and provide the best reperfusion strategy for each patient with STEMI, regional myocardial infarction (MI) systems of care have been established as coordinated networks of hospitals, emergency services, and healthcare professionals within defined geographic regions [64]. Many of these developments in the United States can be attributed to initiatives like the Mission: Lifeline and STEMI Accelerator Programs. Clinical registries, such as the National Cardiovascular Data Registry (NCDR) and similar programs in Western Europe, are essential resources that provide continuous measurement, track critical processes, and identify areas for improvement [65]. However, the transfer of HIC STEMI models to LMIC settings remains constrained by weaker referral architecture, limited ambulance‐based EMS coverage, and fewer resources to sustain coordinated regional systems of care [8]. The prevailing pathways of STEMI care in North America and Europe face hurdles in LMICs [66]. STEMI system models developed in HICs are often difficult to implement in LMIC settings, because formal referral networks and transfer processes are underdeveloped, and hospitals in many developing nations are not explicitly classified as PCI‐capable or non‐PCI‐capable. Limited availability of organized ambulance‐based EMS represents a major structural barrier. As a result, programs such as the American Heart Association Mission: Lifeline, which depend on coordinated prehospital care and quick transfer for primary PCI, are challenging to execute. In a similar vein, resource limitations continue to make it difficult to meet the ACCF door‐to‐balloon (D2B) Alliance goal of achieving D2B times below 90 min. Crucially, in environments with substantial system delays and missing STEMI networks, there are no explicit recommendations for dealing with late STEMI presenters [12]. Additionally, delays frequently occur after FMC, particularly at initial non‐PCI facilities where delayed ECG acquisition, inefficient triage, or referral inefficiencies prolong FMC‐to‐diagnosis and FMC‐to‐reperfusion intervals. In an extensive study of 7312 STEMI patients presenting to non‐PCI hospitals, 67.1% experienced early presentation delays exceeding 120 min from symptom onset to hospital arrival, suggesting substantial delays before FMC. Even after reaching the initial healthcare facility, 31.4% experienced a delay of more than 10 min to the first ECG, reflecting delayed triage and diagnosis at initial contact. These post‐contact system delays substantially prolong total ischemic time, yet are not captured by conventional performance metrics such as D2B time [67]. Finally, weak prehospital and inadequate early triage processes are major contributors to delayed STEMI care in LMICs [68].

## 7 Public Health, Policy, and Implementation Implications

Pre‐hospital delay in STEMI reflects broader health‐system and policy challenges rather than solely individual patient behavior. Limited availability of regional centers of excellence, referral delays, financial constraints, logistic and infrastructural difficulties, poor ambulance services, traffic congestion, limited public awareness, and health literacy are the major hurdles [69]. Therefore, rather than focusing solely on raising individual knowledge, addressing these delays requires population‐level actions to improve health systems. For example, a study found that strengthening EMS engagement has been shown to reduce treatment delays [70]. Regular coordination mechanisms between health system administrators and EMS agencies can facilitate protocol development and improve system integration. Investment in portable ECG equipment with transmission capability for ambulances and receiving hospitals represents a relatively low‐cost intervention with significant potential to improve early diagnosis. The primary challenge lies less in cost and more in sustaining coordination across emergency care systems. Integrating dispatcher‐coordinated taxis or paratransit services into emergency transport networks may provide a scalable strategy to expand emergency access in settings with limited ambulance availability. This will effectively increase coverage and shorten response times without requiring a significant financial investment. In addition, incorporating basic first‐response training within community and dispatch systems may further strengthen early emergency response capacity. In low‐resource settings, this strategy has been shown to improve response performance in line with international standards [71].

To decrease preventable cardiovascular mortality and health disparities, STEMI care should be incorporated within broader NCD strategies and UHC frameworks. Since prompt reperfusion for STEMI is a crucial, life‐saving intervention that cannot be postponed without serious consequences for survival, emergency cardiovascular care should be recognized as an essential health service within national health systems. Integrating acute cardiac care within Sustainable Development Goal 3 (Good Health and Well‐being) aligns emergency response systems with targets on financial risk protection, universal access to care, and reduction of premature NCD mortality. Instead of treating STEMI as a tertiary‐care problem, this integration for LMIC health planning requires giving pre‐hospital systems, referral networks, and financing mechanisms for emergency cardiac treatment top priority within national NCD policies [72]. Furthermore, to treat time‐sensitive illnesses like STEMI within NCD frameworks, the World Health Organization (WHO) also emphasizes the importance of integrating emergency care into health system planning and service delivery to address time‐sensitive conditions [73].

## 8 Discussion

Pre‐hospital delay in STEMI is influenced by interacting individual, systemic, and structural factors. While traditional views often attribute these delays to patient denial or lack of awareness, evidence shows that systemic issues play a substantial role, particularly in LMICs. Important factors include the organization of EMS, geographic accessibility, socioeconomic constraints, and inefficiencies in referral pathways [37, 68].

Beyond summarizing individual determinants, the four‐pathway framework used in this review helps clarify where interventions should be prioritized. While recognition and decision‐making barriers contribute to early delay, improvements in access to FMC and system readiness after contact may yield greater reductions in total ischemic time. This sequencing perspective is particularly relevant in LMIC contexts, where system‐level barriers often outweigh awareness deficits.

Demographic factors such as female sex, older age and diabetes are consistently associated with longer symptom onset–to–FMC intervals. These patterns likely reflect both biological and sociocultural influences on symptom interpretation and care‐seeking behavior [74, 75]. Younger males tend to seek care more quickly, whereas women and older adults may misinterpret nonspecific symptoms or hesitate to activate EMS [75, 76]. Additionally, studies from Portugal indicate that delays are also influenced by patient understanding of symptoms and the decision‐making process, with self‐transport significantly increasing pre‐hospital times [24].

Across settings, the access pathway appears especially important because delayed EMS activation and transport limitations repeatedly amplify the effects of recognition and decision‐making barriers [38, 77, 78, 79].

The benefits of prehospital ECG and other early diagnostic strategies depend heavily on whether patients engage with EMS and whether downstream systems can respond quickly once FMC occurs [26, 51]. This gap highlights the challenges in translating technological advancements into equitable care, especially in LMICs with limited EMS coverage and fragmented referral networks [68].

Differences between HIC and LMIC settings suggest that delays are shaped less by symptom awareness alone and more by how effectively health systems convert symptom recognition into timely first medical contact and definitive care.

Operationalizing an equity lens requires routine reporting of delay intervals stratified by a minimal core set of variables, including sex, age, rural versus urban residence, socioeconomic indicators (e.g., insurance status or area‐level deprivation), and ethnicity, where contextually appropriate. Linking these stratifiers to pathway‐specific barriers, such as atypical symptom recognition in older adults (recognition pathway), financial hesitation among uninsured populations (decision‐making pathway), rural EMS response times (access pathway), and triage inefficiencies at non‐PCI facilities (system readiness pathway), can guide targeted and measurable interventions.

So‐called “invisible delays” often occur after FMC and represent post‐contact system delays. Although these intervals are measurable, they are frequently not incorporated into conventional performance metrics, particularly when patients first present to non–PCI‐capable facilities. Distinguishing symptom onset–to–FMC delay from post‐contact system delay (FMC‐to‐diagnosis and FMC‐to‐reperfusion) is therefore essential for accurately quantifying total ischemic time [80, 81].

Prolonged pre‐hospital delays are consistently associated with adverse outcomes, including reduced eligibility for timely reperfusion and higher morbidity and mortality [38, 77]. Even modest improvements in EMS response capacity can significantly enhance outcomes, which aligns with ACC/AHA and ESC guidelines emphasizing early EMS activation and rapid transport to definitive care [37, 51].

The multifactorial nature of pre‐hospital delay indicates that interventions such as public awareness campaigns alone are insufficient. While patient education remains part of multifaceted strategies, its independent impact is limited in the presence of structural barriers [51, 78]. System‐level interventions, including expanded EMS coverage, integrated pre‐hospital diagnostics and referral networks, subsidized transport and care in LMICs, and embedding emergency care within UHC and NCD programs, are more likely to achieve lasting improvements in timeliness and equity [38, 79, 82]. In settings considering dispatcher‐coordinated taxis or para‐transit vehicles to augment emergency transport capacity, safeguards related to safety standards, training, clinical oversight, accountability and medico‐legal governance are essential to ensure patient protection and system reliability.

### 8.1 System Improvements in LMICs Over the Last Two Decades and Capacity Building Strategies

Despite the structural barriers described above, measurable progress in STEMI care has occurred in several LMIC settings over the past two decades. The government‐sponsored Tamil Nadu STEMI program in India, implemented as a hub‐and‐spoke network using social media‐based ECG transmission and telemedicine consultation, substantially increased guideline‐directed reperfusion and referrals to PCI‐capable centers over a 5‐year evaluation period [3, 15]. The Latin American Telemedicine Infarct Network (LATIN) in Brazil and Colombia connected primary care facilities to round‐the‐clock PCI hubs via expert ECG teleconsultation; among over 300,000 patients remotely screened, nearly half of the diagnosed acute MI cases received urgent reperfusion therapy, compared with historical controls [83]. In Salvador, Brazil, a public regional STEMI network expanded its coverage by 300,000 individuals and increased reperfusion delivery rates from 31% to 73% over a 10‐year period through sustained quality improvement interventions [7]. China's Chest Pain Center initiative improved pre‐hospital diagnostic efficiency and shortened door‐to‐ECG and ECG‐to‐team‐trigger intervals across hundreds of participating hospitals [47].

These experiences highlight several transferable capacity‐building strategies: (i) telemedicine‐supported ECG interpretation and remote catheterization laboratory activation to bridge geographic distances, (ii) task‐shifting of initial STEMI recognition and fibrinolysis administration to trained non‐physician providers at peripheral facilities, (iii) dispatcher‐coordinated first‐response networks, and (iv)mobile messaging platforms for rapid ECG transmission and team activation. Persistent challenges include the sustainability of pilot programs beyond project funding cycles, their scalability to national levels, and the inequitable concentration of gains in urban tertiary centers. Critically, isolated technical improvements are insufficient without supportive governance, financing, and accountability mechanisms. Embedding emergency cardiac care as an essential health service within UHC benefit packages and within national NCD action plans, as called for in the WHO Global Action Plan for the Prevention and Control of NCDs 2013–2030 and aligned with Sustainable Development Goal 3.4, would formalize the political and fiscal commitments required to sustain and scale these improvements [72, 73, 84]. Future research should prioritize implementation science studies evaluating the scalability, cost‐effectiveness, and equity impact of LMIC‐adapted STEMI systems rather than simply replicating HIC models without contextual adaptation [8].

## 9 Limitations

This review has several limitations. As a narrative synthesis, it does not provide pooled quantitative estimates or a formal risk‐of‐bias assessment. Reported delay intervals vary across study designs, populations, reperfusion eras (thrombolysis vs. primary PCI) and endpoint definitions (e.g., symptom onset–to–FMC vs. symptom onset–to–hospital arrival), limiting direct cross‐study comparability and causal inference.

In addition, available literature is largely observational and registry‐based, and may be subject to reporting and publication bias, which can skew the evidence toward settings with better‐developed reporting infrastructure, amplifying apparent HIC–LMIC contrasts. Although efforts were made to ensure structured synthesis, the absence of a formal systematic review protocol and study selection flow diagram may limit reproducibility. These constraints should be considered when interpreting system‐level conclusions.

Heterogeneity across studies warrants particular emphasis. Variation in delay endpoint definitions (symptom onset‐to‐FMC vs. symptom onset‐to‐hospital arrival vs. symptom onset‐to‐first ECG), differences in inclusion criteria and reperfusion era (thrombolysis vs. primary PCI) substantially limit cross‐study comparability. The predominantly observational and registry‐based evidence base introduces additional concerns, self‐reported symptom onset times are subject to recall bias, registry participation and data quality vary substantially across settings, and selection bias may systematically underrepresent the most disadvantaged populations, including those with the longest delays who never reach a facility.

Randomized controlled trials addressing pre‐hospital delay reduction or EMS system interventions in LMIC contexts remain rare, and policy trials evaluating the impact of governance or financing changes on delay intervals are virtually absent from the published literature. This gap between descriptive epidemiology and implementation evidence substantially constrains the ability to make causal inferences or evidence‐graded policy recommendations.

The predominance of descriptive evidence in this field highlights the need for stronger implementation research, prospective cohort studies embedded within quality‐improvement programs, quasi‐experimental evaluations of system‐level interventions, and health economic analyses of context‐appropriate STEMI network models. Future research should report delay data stratified at a minimum by sex, age, geographic location, and socioeconomic status to enable equity‐sensitive synthesis. Adherence to established reporting standards, such as SQUIRE for quality improvement research and STROBE for observational studies, would improve cross‐study comparability. Alignment with current ACC/AHA and ESC STEMI guidelines, which call for comprehensive emergency cardiac care systems and explicit pre‐hospital activation protocols, should guide the design of future implementation studies and policy evaluations [13, 84].

## 10 Conclusion

Pre‐hospital delay in STEMI reflects a complex interplay of recognition barriers, decision‐making dynamics, access constraints, and system readiness limitations. In HICs, further gains are likely to come from strengthening EMS activation, reducing inequities in early presentation, and maintaining coordinated diagnostic and reperfusion systems. In LMICs, the greatest gains will likely come from foundational investments in emergency access, first‐contact diagnostic capacity, referral coordination, and financial protection. Prioritizing scalable, pathway‐specific strategies offers a pragmatic approach to reducing avoidable ischemic time and narrowing global disparities in STEMI outcomes (Tables 1 and 2).

**Table 1:** Summary of key determinants of pre‐hospital delay by income setting.

Determinant/Factor	Description/Mechanism	In HICs (typical pattern)	In LMICs (typical pattern)	Key differences/magnitude
1. Symptom recognition & interpretation	Misattribution (e.g., to gastric issues), atypical presentations	Moderate residual delay; advanced EMS, pre‐hospital ECG, and system optimization reduce it	Prolonged delay; frequent misattribution, atypical symptoms unrecognized	Much longer in LMICs; rural/geographical factors amplify
2. Atypical presentations	Women, the elderly, diabetics often have non‐classic symptoms	Persistent but mitigated by targeted improvement/EMS	Severe under‐recognition; longer delays in women/elderly	Stronger gender/age/ethnic/racial inequities in LMICs
3. Family‐mediated decision‐making	Household involvement in deciding to seek care	Less dominant; more individual autonomy	Very strong; gender/power dynamics delay action	Major barrier in LMICs
4. Anticipated cost/financing barriers	Fear of expenses delaying care‐seeking	Minimal	Major; out‐of‐pocket payments, insurance gaps	Central driver in LMICs
5. EMS availability & reliability	Access to ambulance, pre‐hospital ECG, fast response	Widespread, advanced (pre‐hospital diagnosis common)	Limited/fragmented; often non‐existent or slow	Structural failure in LMICs
6. Transport preferences & geographic constraints	Private vs public transport; urban‐rural divide	Moderate rural delay; good roads/infrastructure	Severe; long distances, poor roads, preference for private	Geography as equity issue in LMICs
7. Referral efficiency & mis‐triage	Waiting at first facility, wrong triage, bottlenecks	Streamlined in most systems	Frequent “invisible delays”; mis‐triage common	Major hidden contributor in LMICs
8. Policy/governance/financing environment	Overall system readiness, funding, regulation	Strong, well‐resourced	Fragmented, under‐funded	Core reason HIC interventions underperform in LMICs
9. Symptom recognition & interpretation	Misattribution (e.g., to gastric issues), atypical presentations	Moderate residual delay; advanced EMS, pre‐hospital ECG, and system optimization reduce it	Prolonged delay; frequent misattribution, atypical symptoms unrecognized	Much longer in LMICs; rural/geographical factors amplify
10. Atypical presentations	Women, elderly, diabetics often have non‐classic symptoms	Persistent but mitigated by targeted improvement/EMS	Severe under‐recognition; longer delays in women/elderly	Stronger gender/age/ethnic/racial inequities in LMICs
11. Family‐mediated decision‐making	Household involvement in deciding to seek care	Less dominant; more individual autonomy	Very strong; gender/power dynamics delay action	Major barrier in LMICs
12. Anticipated cost/financing barriers	Fear of expenses delaying care‐seeking	Minimal	Major; out‐of‐pocket payments, insurance gaps	Central driver in LMICs
13. EMS availability & reliability	Access to ambulance, pre‐hospital ECG, fast response	Widespread, advanced (pre‐hospital diagnosis common)	Limited/fragmented; often non‐existent or slow	Structural failure in LMICs
14. Transport preferences & geographic constraints	Private versus public transport; urban‐rural divide	Moderate rural delay; good roads/infrastructure	Severe; long distances, poor roads, preference for private	Geography as equity issue in LMICs
15. Referral efficiency & mis‐triage	Waiting at the first facility, wrong triage, bottlenecks	Streamlined in most systems	Frequent “invisible delays”; mis‐triage common	Major hidden contributor in LMICs
16. Policy/governance/financing environment	Overall system readiness, funding, and regulation	Strong, well‐resourced	Fragmented, under‐funded	Core reason HIC interventions underperform in LMICs

*Note:* See main text Section 5 for details.

Abbreviations: HICs = high‐income countries, LMICs = low‐ and middle‐income countries.

**Table 2:** Mapping of intervention strategies and implementation gaps.

Intervention strategy	Effectiveness in HICs	Effectiveness in LMICs	Main implementation gaps in LMICs	Feasibility/Scalability in LMICs
Public awareness & education campaigns	Moderate reduction in delay; limited long‐term impact	Limited/short‐term effect; poor sustained behavior change	Low health literacy, cultural barriers, lack of public education infrastructure	Medium
EMS expansion & pre‐hospital ECG/procedures	Strong; declining symptom‐to‐door times, faster reperfusion via pre‐hospital ECG, Cath‐lab activation, standardized procedures	Partial/inconsistent; major delays persist	Absence of organized regional STEMI systems, limited financial/infrastructural/human resources, underdeveloped EMS, no pre‐hospital ECG capacity	Low–Medium
Pre‐hospital Cath‐lab activation/diagnosis	Effective; improves in‐hospital outcomes, shorter system delays	Very limited/rarely implemented	Lack of PCI‐capable hospital classification, missing ambulance‐based EMS, and no coordinated prehospital care	Low
Regional STEMI networks/systems of care	Significant improvement in outcomes; integrated facilities & professionals (e.g., Mission: Lifeline, STEMI Accelerator)	Suboptimal/largely absent	Antiquated healthcare systems, implementation & knowledge gaps, insufficient resources to build/maintain registries & networks	Low
Community first‐responder models & dispatcher‐coordinated transport	Emerging benefit; effective in shortening system times	Promising but under‐evaluated; scalable in low‐resource settings	Training sustainability, volunteer retention, coordination with existing (often absent) EMS	Medium–High
Task‐shifting & decentralization of emergency care	Limited data in HICs	High potential; improves response in low‐resource settings	Regulatory barriers, quality control, and inadequate emergency training	High
Low‐cost transport & referral innovations	N/A (not primary in HICs)	Emerging; can increase coverage & shorten response times	Traffic congestion, poor coordination, safety concerns, and lack of formal integration	High
Integration into NCD/UHC frameworks & policy prioritization	N/A (already embedded in HIC systems)	High potential; aligns with SDG 3 & WHO recommendations	Insufficient prioritization of pre‐hospital/referral/financing in national NCD policies	High

*Note:* See main text Section 6 for details.

Abbreviations: HICs = high‐income countries, LMICs = low‐ and middle‐income countries.

## Funding

The authors have nothing to report.

## Conflicts of Interest

The authors declare no conflicts of interest.

## Data Availability

Data sharing not applicable to this article as no datasets were generated or analyzed during the current study. All data analyzed in this narrative review were obtained from previously published studies that are publicly available and cited in the references. No new primary data were generated.
